# Renal Dopamine Receptors, Oxidative Stress, and Hypertension

**DOI:** 10.3390/ijms140917553

**Published:** 2013-08-27

**Authors:** Santiago Cuevas, Van Anthony Villar, Pedro A. Jose, Ines Armando

**Affiliations:** Department of Medicine, Division of Nephrology, University of Maryland School of Medicine, 20 Penn St., HSFII, Suite S003, Baltimore, MD 21201-1599, USA; E-Mails: vvillar@medicine.umaryland.edu (V.A.V.); pjose@medicine.umaryland.edu (P.A.J.); iarmando@medicine.umaryland.edu (I.A.)

**Keywords:** dopamine receptors, oxidative stress, kidney, hypertension

## Abstract

Dopamine, which is synthesized in the kidney, independent of renal nerves, plays an important role in the regulation of fluid and electrolyte balance and systemic blood pressure. Lack of any of the five dopamine receptor subtypes (D1R, D2R, D3R, D4R, and D5R) results in hypertension. D1R, D2R, and D5R have been reported to be important in the maintenance of a normal redox balance. In the kidney, the antioxidant effects of these receptors are caused by direct and indirect inhibition of pro-oxidant enzymes, specifically, nicotinamide adenine dinucleotide phosphate, reduced form (NADPH) oxidase, and stimulation of anti-oxidant enzymes, which can also indirectly inhibit NADPH oxidase activity. Thus, stimulation of the D2R increases the expression of endogenous anti-oxidants, such as Parkinson protein 7 (PARK7 or DJ-1), paraoxonase 2 (PON2), and heme oxygenase 2 (HO-2), all of which can inhibit NADPH oxidase activity. The D5R decreases NADPH oxidase activity, via the inhibition of phospholipase D2, and increases the expression of HO-1, another antioxidant. D1R inhibits NADPH oxidase activity via protein kinase A and protein kinase C cross-talk. In this review, we provide an overview of the protective roles of a specific dopamine receptor subtype on renal oxidative stress, the different mechanisms involved in this effect, and the role of oxidative stress and impairment of dopamine receptor function in the hypertension that arises from the genetic ablation of a specific dopamine receptor gene in mice.

## 1. Introduction

The redox state of cells can be defined as the steady state condition whereby the generation of free radical/highly reactive species is balanced by antioxidant mechanisms. Reactive oxygen species (ROS) normally act as cellular messengers. Enzymes that produce ROS are present in subcellular compartments, including lipid rafts, such that ROS levels can increase to a critical level for signal transduction and then be destroyed in a timely manner [1,2]. ROS are also involved in the destruction of invading pathogens and low levels of ROS may increase life span (hormesis) [3]. However, increased oxidative stress and its failure to return to initially normal levels disrupt normal cellular signaling mechanisms [4–6]. The disturbance in the normal redox state of cells results in oxidative stress, a condition characterized by an overproduction of free radicals that are toxic to the cell.

Several families of enzymes and receptors are involved in the regulation of redox balance, including the nicotinamide adenine dinucleotide, reduced form (NADPH) oxidase, and the dopamine receptors. The Nox family of NADPH oxidases is comprised of enzymes that couple electrons from NADPH to molecular oxygen to generate superoxide. There are seven Nox homologs, four of which (Nox1, Nox2, Nox4, and Nox5) are found in the vasculature and the kidney where they constitute the major sources of ROS [7,8]. Increased Nox activity boosts the production of ROS that participate in the pathogenesis of several disorders, including hypertension [7–9]. For example, Nox1, Nox2, and Nox4 are increased in several tissues in rats with spontaneous hypertension or angiotensin II-induced hypertension [10–13]. However, the role of Nox4 in hypertension is not entirely clear because *Nox4* knockout mice are normotensive [9]. The protein expression of the Nox5 gene, which is present in humans but not rodents, is greater in renal proximal tubular cells from hypertensive than normotensive humans, and may account for the increased oxidative stress in renal proximal tubule cells from hypertensive humans [14]. Several studies have shown that NADPH oxidase [15,16], by direct and indirect mechanisms, can be positively regulated by ROS, causing a positive “feedback loop” that may trigger the development of diseases such as hypertension. However, oxidative stress has yet to be established as a cause of human essential hypertension. Species specificity has to be kept in mind. For example, the role of lipid rafts in the production of ROS is species-specific; in renal proximal tubule cells, lipid rafts keep NADPH oxidase in the active state in rats but keep NADPH oxidase in the inactive state in humans [17,18].

### 1.1. Renal Dopaminergic System

Dopamine is synthesized by the kidney, mainly by renal proximal tubule cells, independent of renal nerves. Unlike in neural tissue dopamine synthesized by renal tubules is not converted to norepinephrine. Renal dopamine is crucial in the maintenance of normal fluid, electrolyte balance, and redox balance and blood pressure [19]. The importance of renal endogenous dopamine in body homeostasis is demonstrated in genetically altered mice with decreased or increased renal dopamine production. The selective deletion in the mouse renal proximal tubule of aromatic amino acid decarboxylase (AADC), the enzyme responsible for the production of dopamine in the kidney, decreased intrarenal dopamine levels, and caused salt-sensitive hypertension [20]. Deletion of catechol-*O*-methyl transferase (COMT), which degrades dopamine to 3-methoxytyramine, is associated with increased dopamine levels. Transplanting the kidney from COMT^−/−^ mice into diabetic wild-type mice ameliorated the consequences of diabetes, decreasing albuminuria, glomerulopathy, inflammation, oxidative stress, and fibrosis, effects that were aggravated by the proximal tubular deletion of AADC [21].

Dopamine exerts its actions via two subfamilies of G protein-coupled receptors (GPCRs), namely, the D1-like dopamine receptors (D1R and D5R subtypes) and D2-like dopamine receptors (D2R, D3R, and D4R subtypes). All the dopamine receptor subtypes are differentially expressed along the nephron, from the proximal tubule to the collecting duct. The D1-like receptors couple to Gαs and stimulate adenylyl cyclases, while the D2-like receptors couple to Gαi and Go, inhibit adenylyl cyclases and calcium channels, and modulate potassium channels. Dopamine also stimulates renal prostaglandin synthesis via D2-like rather than D1-like receptors [19]. Both subfamilies of dopamine receptors link to MAPK activation, although through different pathways. Dopamine receptors may also exert their actions by inhibiting signaling pathways. Thus, the D2R can suppress Akt (protein kinase B) signaling [22]. The dopamine receptors interact among themselves, resulting in new signaling pathways that are probably cell specific. For example, in neurons, the interaction between D1R and D2R can lead to stimulation of phospholipase C [23]. However, D1R, independent of D2R, can stimulate phospholipase Cβ1 in renal cortical cells [24] and phospholipase Cγ in fibroblasts [25].

### 1.2. Reactive Oxygen Species

ROS are important in the pathogenesis of hypertension and the increase in ROS production and blood pressure, caused by stimulation of the renin-angiotensin-aldosterone system, is well known. However, the negative regulators of ROS production are not well understood. Some downstream negative regulators of ROS, e.g., epoxyeicosatrienoic acids, are known but the negative regulators upstream to these pathways are relatively unknown. One such upstream negative regulator, under physiological conditions, may be the renal dopaminergic system [19]. Intrarenal dopamine counteracts the oxidative stress in deoxycorticosterone acetate/high salt-induced hypertension and angiotensin II-mediated renal injury [26]. Dopamine counteracts oxidative stress not only by inhibiting pro-oxidant enzymes, e.g., NADPH oxidase, but also by stimulating antioxidant enzymes, e.g., extracellular superoxide dismutase (SOD), heme-oxygenase (HO). However, dopamine, at high concentrations (≥10 μM) [27,28], can lead to increased ROS production as a consequence of auto- or enzymatic oxidation [29,30].

In this review, we provide an overview of the role of each of the dopamine receptor subtypes in the regulation of renal oxidative stress, the mechanisms involved in these effects, and the participation of oxidative stress in the hypertension that develops in dopamine receptor knockout mice.

## 2. Renal Dopamine Receptor Regulation of Oxidative Stress

### 2.1. D1-Like Receptors

#### 2.1.1. Renal Expression of D1-Like Dopamine Receptors

The D1-like dopamine receptors, D1R and D5R, are expressed in the apical and basolateral membranes of the proximal tubule, medullary thick ascending limb of Henle, distal convoluted tubule, and cortical collecting duct [19,31–33]. They are not found in the glomerulus [19], although they are expressed in mouse glomerular podocytes in culture [34]. D1R, but not D5R, is expressed in the macula densa and juxtaglomerular cells in rats [35]. The expression of either D1R or D5R has not been reported in macula densa or juxtaglomerular cells in the human or mouse kidney [33]. Both D1R and D5R receptors are present in the small and large intrarenal arteries in rodents and humans [32,33,36,37].

Renal D1-like receptors are major physiological regulators of epithelial sodium transport and the lack of the expression or function of these receptors is associated with renal retention of sodium and increased blood pressure [19,38].

#### 2.1.2. D1-Like Receptors Negatively Regulate ROS Production

As with high concentrations of dopamine, high concentrations of D1-like receptor agonist (100 μM, SKF38393) [39] can also increase ROS production. However, low concentrations of D1-like receptor agonists decrease oxidative stress in many cells, including lymphocytes, and brain cortical, vascular smooth muscle, and renal proximal tubule cells [17–19,40–42], by decreasing the production of ROS [17–19,40–42] and reactive nitrogen [43]. Several signaling pathways are involved in the antioxidant effects of D1-like receptors. In retinal ganglion cells, D1-like receptor stimulation attenuated hydrogen peroxide-induced injury via the ERK and p38 pathways [44]. In vascular smooth muscle cells, D1-like receptor agonists inhibited platelet-derived growth factor-BB-mediated oxidative stress through activation of protein kinase A (PKA), and suppression of phospholipase D (PLD) and protein kinase C (PKC). The PKA inhibitor H-89 reduced the antioxidant effect of dopamine in rat vascular smooth muscle cells, effects that were blocked by treatment with antisense oligonucleotides to either D1-like receptor subtype, indicating that in these cells the two D1-like receptor subtypes mediate the antioxidant effect of dopamine [41,45].

The antioxidant effects of D1-like receptors are eventually exerted by inhibiting the pro-oxidant enzyme, NADPH oxidase, and stimulating the anti-oxidant enzyme, heme oxygenase-1 (HO-1) [19]. The ability of D1-like receptors to stimulate antioxidant enzymes such as glutathione peroxidase, SOD-1, and glutamylcysteine transferase, involves Nrf-2 [46]. However, the pathways involved in these effects may be specific to D1R or D5R. Studies in HEK293 cells heterologously expressing either the human D1R or the human D5R have shown that the pathway involved in the inhibition of NADPH oxidase activity is D1-like receptor specific. Thus, the inhibition of NADPH oxidase by the D1R is mediated by stimulation of PKA and PKC cross-talk [47]. By contrast, the D5R decreases NADPH oxidase activity via inhibition of PLD [43] and activation of HO-1 [48]. Mice lacking D1R or D5R are hypertensive, highlighting the importance of these receptors in the regulation of blood pressure. Although the redox status of *D1R*^−/−^ mice remains to be determined, the hypertension in *D5R*^−/−^ mice is associated with increased oxidative stress related to increased pro-oxidant and decreased antioxidant activity [43,48,49]. *D5R*^−/−^ mice have high levels of plasma thiobarbituric acid reactive substances, a byproduct of lipid peroxidation, and renal Nox2 (gp91phox) and Nox1 (p47phox) expression. Nox activity is also increased in the brain and kidney in *D5R*^−/−^ mice compared to their wild-type littermates, and chronic treatment with apocynin (an NADPH oxidase inhibitor) ameliorated the increased blood pressure, plasma thiobarbituric acid reactive substances, and NADPH activity in the brain and kidney of *D5R*^−/−^ mice [49]. In contrast, the renal expression of HO-1 is decreased in *D5R*^−/−^ mice [48,49]; these mice have increased α/β hydrolase 1 mRNA expression, possibly as a compensatory mechanism to ameliorate the increased NADPH activity [50].

Both D1R and D5R exert additional antioxidant effects by their negative regulation of the expression and function of the angiotensin II type 1 receptor (AT1R) [19], which can increase ROS production, in part, by activation of NADPH oxidase via PLC, PKC, and calcium signaling [51,52]. In rat renal proximal tubule cells the D1R inhibits AT1R function by different mechanisms depending on the duration of exposure. In the short-term (min), D1R causes a rapid partial internalization of the AT1R and complete abolition of AT1R signaling [53], while in the long-term (24 h), it decreases the total abundance of the AT1R receptor [54,55]. D5R stimulation increases the degradation of glycosylated AT1R in proteasomes in human renal proximal tubule cells consequently decreasing AT1R protein abundance [56]. Furthermore, the renal expression of AT1R is increased in *D5R*^−/−^ mice relative to wild-type littermates [56,57] indicating that in the basal state the constitutively active D5R decreases AT1R expression. However, decreased D1R and increased AT1R function does not necessarily cause an increase in ROS production as demonstrated in G protein-coupled receptor kinase (GRK)4γ^142V^ transgenic mice which have increased blood pressure and AT1R function, as well as decreased D1R function, but normal ROS production, probably related to increased HO-1 expression [58]; hGRK4γ^142V^ desensitizes the D1R but not the D5R [19,38].

#### 2.1.3. ROS Negatively Regulate D1-Like Receptor Expression and Function

While D1-like receptors inhibit ROS production [19,38,42,43,45–50], the function of these receptors is impaired by oxidative stress. Renal proximal tubule cells from old rats, which have increased oxidative stress, have decreased expression of D1R [59]. Treatment with tempol, an SOD mimetic, or exercise, reduced the renal oxidative stress and normalized renal D1R expression and function in old rats [60,61]. Decreasing the oxidative stress also restored the D1R coupling to G-proteins in obese Zucker [62] and streptozotocin-treated hyperglycemic rats [63]. In obese Zucker and streptozotocin-treated hyperglycemic rats, treatment with tempol restored the D1R responses and normalized the blood pressure in obese Zucker rats [62]; streptozotocin-treated hyperglycemic rats were normotensive and their blood pressures were not affected by tempol [63]. Sprague-Dawley rats, fed high salt diet and the oxidant l-buthionine sulfoximine (glutathione synthesis inhibitor) had increased blood pressure and impaired D1R function; these effects were prevented by treatment with tempol [64]. The impairment of D1R function by oxidative stress (e.g., H_2_O_2_) in renal proximal tubule cells was mediated by the nuclear translocation of nuclear factor kappa-light-chain-enhancer of activated B cells (NF-κB), activation of PKC, and translocation of GRK2 to the plasma membrane, which, in turn, caused D1R hyper-serine phosphorylation and uncoupling, thus, impairing its activity [46,65–67]. In obese Zucker rats, the D1R hyper-serine phosphorylation and uncoupling in renal proximal tubule cells was related to increased plasma membrane expression of GRK2 and total cellular expression of GRK4 [68]. GRK2 and GRK4 can impair the function of D1R [19,38].

### 2.2. D2-Like Receptors

#### 2.2.1. Renal Expression of D2-Like Dopamine Receptors

D2 like dopamine receptors, D2R, D3R, and D4R, are all expressed in the kidney [19]. D2R is expressed in the proximal tubule, thick ascending limb, distal convoluted tubule, and cortical collecting duct in mice, rats, and humans. D2R is also expressed in the outer medullary collecting duct in rats and humans but not in mice and inner medullary collecting duct in rats but not in mice or humans. Glomerular mesangial cells in rats and glomerular podocytes in humans also express D2R. D2R is not expressed in juxtaglomerular cells but may be expressed in macula densa cells in rats [19,69]. The long form (D2LR), rather than the short form (D2SR) of D2R, is expressed in the renal tubule [70].

D3R is expressed in the proximal tubule and distal convoluted tubule in rodents, as well as in the thick ascending limb in mice but not rats while it is expressed in the cortical collecting duct in rats but not mice. D3R is also expressed in mesangial cells and podocytes, juxtaglomerular cell and macula densa, and arterial vessels in rodents. D3R is expressed in human renal proximal tubule cells; D3R expression in other segments of the human nephron has not been reported [19]. In the rat renal proximal tubule, D3R protein is mainly located in the apical and subapical areas [71].

D4R is expressed in proximal and distal convoluted tubules [72], thick ascending limb, and cortical and outer medullary collecting ducts [69,73] in rodents. D4R is also expressed in the macula densa in rats but not mice [19,74]. D4R is expressed in arterial vessels but not glomeruli in rodents. D4R protein expression in the human kidney has not been reported although D4R mRNA is expressed in the human kidney [75].

Dopamine D2-like receptors can be vasodilator or vasconstrictor, and natriuretic or antinatriuretic [19,76,77], depending on renal nerve activity or the state of sodium balance. D2-like receptor agonists also have antioxidant effects at low concentrations and have direct and/or indirect protective effects *in vivo* and *in vitro* via their antioxidant effects [78]. Ropinirole, a D2R/D3R/D4R agonist, which has the highest affinity for D2R among D2-like receptors, scavenged free radicals, suppressed lipid peroxidation but increased glutathione, catalase, and SOD activities in the striatum, and protected striatal dopaminergic neurons against 6-hydroxydopamine injury in mice. Pre-treatment with sulpiride, a D2R/D3R antagonist, prevented the antioxidant and neuroprotective effects of ropinirole [79].

#### 2.2.2. D2R Negatively Regulates ROS Production

D2R agonists have neuroprotective effect against oxidative stress and scavenge free radicals [79–81], although high concentrations of D2R agonist (10 μM raclopride) [82], as with D1-like receptor agonists, can also increase ROS production. In cultured rat mesencephalic neurons, pre-incubation with low concentrations of D2-like dopamine receptor agonists provided neuroprotection against glutamate-induced oxidative stress. *In vivo* and *in vitro* studies have also shown that the protective effects of D2R agonists are abolished in the presence of D2R antagonists, indicating D2R specificity [83,84]. By contrast, D2R antagonists can induce oxidative damage in the brain. Adult male Wistar rats treated with haloperidol had increased ROS production in the striatum and protein carbonyls in the hippocampus [81]. Stimulation of the D2R in neurons from rat embryonic ventral mesencephalon was protective of levodopa toxicity [84] and in mouse or human renal proximal tubule cells decreased ROS production, Nox4 expression, and NADPH oxidase activity [19,85,86].

##### 2.2.2.1. D2R Protects against Oxidative Stress: Role of NADPH Oxidase

A protective role of the D2R against oxidative stress was also uncovered in mice lacking D2R (*D2R*^−/−^). These mice have high blood pressure [19,87], are salt-sensitive [87], and have increased oxidative stress [85,86,88], proved by increased urinary excretion of 8-isoprostane and renal expression of Nox isoforms and activity of NADPH oxidase. Apocynin normalized the elevated blood pressure in *D2R*^−/−^ mice. Spironolactone also normalized the high blood pressure of *D2R*^−/−^ mice but did not normalize the renal expression of NADPH oxidase, indicating that the increased ROS production was only partly mediated by impaired aldosterone regulation [88] and that, in this model, increased ROS were involved in the development or maintenance of high blood pressure.

##### 2.2.2.2. D2R Protects against Oxidative Stress: Role of the Antioxidant, DJ-1

The regulation of ROS production by the D2R involves not only the inhibition of pro-oxidant systems (e.g., NADPH oxidase, vide supra) but also the stimulation of antioxidant systems [85,86,88]. D2R^−/−^ mice have decreased renal expression of the antioxidant enzyme HO-2 in the kidney. HO-2 can inhibit NADPH oxidase activity [89]. The antioxidant effect of D2R also involves its interaction with two other proteins, DJ-1 (also known as Park 7) and paraoxonase 2 (PON2). DJ-1 is a protein originally described as an oncogene and identified as an autosomal-recessive gene of Parkinson disease [90]. DJ-1 is expressed in several rodent and human tissues, such as the brain, heart, kidney, liver, pancreas, and skeletal muscle [90] and its protective role against oxidative stress has been demonstrated in several disease states [91–93]. DJ-1 has intrinsic antioxidant activity as it is an atypical peroxiredoxin-like peroxidase that scavenges H_2_O_2_ through oxidation of Cys-106 [94] and also regulates the expression of several antioxidant genes, such as SOD [95–97]. In the absence of oxidative stress, DJ-1 binds to and represses the translation of antioxidant factors, such as SOD, and proteins involved in glutathione synthesis [98–100]. By contrast, oxidized DJ-1 dissociates from these transcripts, allowing their translation [100]. DJ-1 expression modulated astrocyte-mediated protection against neuronal oxidative stress [101] and lack of DJ-1 impaired astrocyte-mediated neuroprotection [102]. Although DJ-1 protects against neurotoxicity [103], *DJ-1*^−/−^ mice did not display increased vulnerability to inflammation-related nigral degeneration, in spite of decreased antioxidant response [104]. In the brain the loss of DJ-1 function resulted in the attenuation of D2R-mediated responses without any change in receptor expression suggesting that the antioxidant effect of DJ-1 is downstream of D2R activation [105]. However, the precise function of DJ-1 in neuronal responses downstream of D2R activation remains to be defined.

DJ-1 is highly expressed in normal heart tissue. DJ-1-deficient mice subjected to oxidative stress had cardiac hypertrophy and increased susceptibility to developing heart failure [106]. DJ-1 has also been reported to protect mouse erythroid cells [107] and pancreatic β-cells [108] from oxidative damage. Indeed, oxidative stress caused by high concentrations of dopamine (≥50 μM) has been reported to increase DJ-1 expression [28].

D2R and DJ-1 colocalize and coimmunoprecipitate in the mouse kidney. Mice with deletion of one D2R allele (*D2*^+/−^) or with selective renal D2R silencing are hypertensive and have increased ROS production and renal cortical expression of Nox4 but decreased expression of DJ-1 [85]. D2R can positively regulate DJ-1 expression [85] but the mechanisms by which D2R regulates the expression of DJ-1 are unknown. The negative regulation of ROS by D2R may be related to its positive regulation of DJ-1 as selective renal silencing of DJ-1 expression in mice increased NADPH oxidase activity and blood pressure [85].

##### 2.2.2.3. D2R Protects against Oxidative Stress: Role of the Antioxidant, PON2

The antioxidant effect of D2R is mediated not only by DJ-1 but also by paraoxonase (PON). The PON family consists of three genes: *PON1*, *PON2*, and *PON3*. PON2 is cell associated, not found in plasma, and expressed in a variety of tissues, including the kidney, and protects against oxidative stress that may be related to hydroperoxidase activity [109–111]. Mouse peritoneal macrophages from *PON2*^−/−^ mice were reported to have increased susceptibility to urokinase plasminogen activator-induced oxidative stress [112]. Overexpression of PON2 prevented apoptosis in vascular endothelial cells [113] and inhibited the development of atherosclerosis in mice [111,114,115], via antioxidant mechanisms. Overexpression of PON2 also inhibited cell-mediated low-density lipoprotein oxidation [116]. It should be noted, however, that ROS can also increase D2R mRNA and protein expression [117], an example of negative feedback inhibition.

D2R colocalized and coimmunoprecipitated with PON2 in brush border membranes of proximal tubules of mouse kidney. Renal D2R can regulate PON2 expression because PON2 mRNA and protein expression were increased by D2R stimulation [86]. Conversely, mice lacking D2R from germline deletion or from renal selective downregulation of the gene had decreased renal PON2 protein expression. The antioxidant effect of D2R was partially prevented by downregulation of PON2 indicating its participation in the antioxidant effect of the D2R. Indeed, silencing PON2 increased the expression of Nox2 and Nox4, and NADPH oxidase activity, and completely abolished the inhibitory effect of a D2R agonist on Nox2 and Nox4 expression [86]. The increase in NADPH oxidase activity with renal silencing of DJ-1 was associated with increased ROS production and blood pressure. Therefore, the positive regulation of PON2 by D2R mediates, with DJ-1, the inhibitory effect of renal D2R on NADPH oxidase activity and ROS production. However, the mechanisms by which DJ-1 and PON2 regulate NADPH oxidase are not yet clear. It is also not known if DJ-1, PON2, and HO-2 interact but we do know that DJ-1 and PON2 do not physical interact in the kidney.

##### 2.2.2.4. D2R Protects against Oxidative Stress and Inflammation

Oxidative stress has been extensively linked to inflammation and vice versa [118]. It is known that stimulation of NF-κB, an important pro-inflammatory transcription factor, increases intracellular ROS production [119]. D2R agonists increased the secretion of anti-inflammatory cytokines by *de novo* gene expression in resting T lymphocytes [120], but suppressed their production in activated T and mast cells [121]. Silencing the D2R in mouse renal proximal tubule cells increased NF-κB transcriptional activity, tumor necrosis factor α (TNFα), and monocyte chemoattractant protein-1 (MCP-1) levels. Selective unilateral renal D2R down-regulation in mice, in the absence of elevated blood pressure, reproduced the alterations in inflammatory factors and renal injury observed in *D2R*^−/−^ mice, increasing the expression of several pro-inflammatory cytokines such as, TNFα, MCP-1, and MCP-2, suggesting that D2R plays a protective role against the development of renal inflammation [122]. Several human polymorphisms of D2R associated with decreased expression or function of the receptor [123,124] have been associated with essential hypertension [125,126], but further studies are needed to determine if the experimental evidence for the role of the D2R in the regulation of oxidative stress in hypertension can be translated to humans.

### 2.3. D3R and Oxidative Stress

The effect of D3R on ROS production is controversial. The D3R has been reported to increase an endogenous factor that has antioxidant actions and thus the D3R may have antioxidant effects, albeit indirectly [127]. Pramipexole, a selective D3R agonist, protected against free-radical induced cytotoxicity, inhibited lipid peroxidation in neurons [128], increased the activity of antioxidant enzymes (glutathione peroxidase and catalase) and inhibited the production of ROS by the mitochondria, however, these effects were independent of D3R activation [129,130]. In contrast, pretreatment with D-264, a D3R agonist, prevented neurotoxin- and lactacystin-induced neurodegeneration, effects that were lost in the presence of a D3R antagonist [131], indicating that the D3R has antioxidant effects.

Disruption of the D3R gene in mice (*D3R*^−/−^) caused renin-dependent hypertension that was associated with decreased ability to excrete an acute intravenous and chronic dietary salt load [132]. However, the hypertension and salt sensitivity in *D3R*^−/−^ mice were not associated with increased renal oxidative stress as demonstrated by normal renal expression of Nox isoforms and nitrotyrosine, as well as normal urinary excretion of 8-isoprostane. The expression of renal D5R, which has antioxidant property [43,45,48,49], was increased in *D3R*^−/−^ mice suggesting that compensatory mechanisms may be involved in maintaining a normal production of ROS in these mice [133].

### 2.4. D4R and Oxidative Stress

Some of the neuroprotective effects of D4R agonists are apparently independent of any action on ROS [134]. However, the D4R antagonist L-745,870 has been reported to decrease the vulnerability of neuronal and non-neuronal cells to oxidative stress-induced apoptosis [135]. By contrast, the activation of D4R has been reported to protect against hypoxia/reoxygenation-induced oxidative stress and cell death in HT22 cells derived from mouse hippocampal neurons [136]. In addition, the protective effects of dopamine and D4R agonist on glutamate-induced ROS production were antagonized by a D4R antagonist [137]. However, mice lacking D4R have increased blood pressure, in part caused by increased AT1R expression [138], but there was no evidence for an increase in oxidative stress in these mice.

## 3. Summary

**Figure 1:**
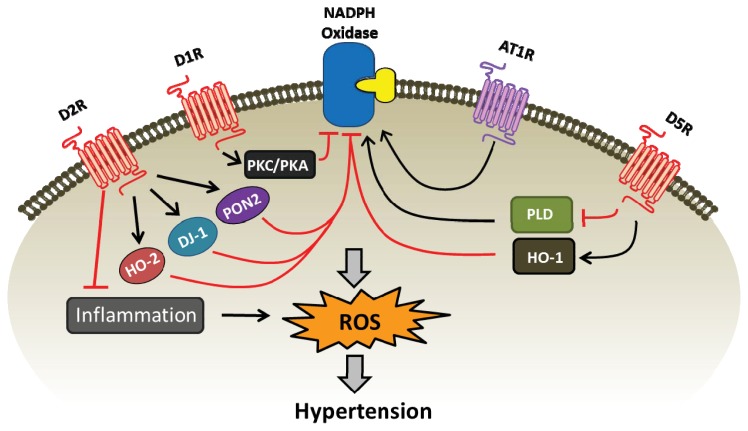
D1R, D2R, and D5R inhibit NADPH oxidase activity and decrease ROS production in the kidney. D1R inhibits NADPH oxidase activity via PKC/PKA pathway (and pathways similar to D5R, see Table 1). D5R inhibits the expression of PLD2 and therefore, NADPH oxidase and increases the expression of HO-1, which also inhibits NADPH oxidase activity. D2R increases HO-2, DJ-1, and PON2 expression, all of which inhibit NAPDH oxidase activity. D2R inhibits TNFα expression and NFκB activity. HO-1, HO-2, DJ-1, and PON2 have antioxidant properties. AT1R increases NADPH oxidase activity. D3R and D4R do not directly affect ROS production.

Physiological concentrations of dopamine have protective effects on oxidative stress in the kidney. D1R, D2R, and D5R inhibit NADPH oxidase activity and ROS production and are needed to keep a normal redox balance (Table 1, Figure 1). D1R inhibits NADPH oxidase activity via PKA and PKC cross-talk and stimulates SOD, glutathione peroxidase, and glutamylcysteine transferase. The D5R decreases NADPH oxidase activity, in part by inhibiting PLD2 and increasing the expression of HO-1, an antioxidant. The D2R also decreases ROS production by increasing the expression of the antioxidants DJ-1, PON2, and HO-2. Lack of any of the dopamine receptor subtypes results in increased blood pressure that is not always associated with increased oxidative stress. Whether or not D3R and D4R protect against oxidative stress remains to be determined; the hypertension in mice lacking D3R and D4R is not associated with oxidative stress.

## Figures and Tables

**Table 1:** Dopamine receptor subtypes D1R, D2R, and D5R regulate the production of reactive oxygen species by inhibiting pro-oxidant and stimulating antioxidant enzymes.

Dopamine receptor subtype	Pro-oxidant enzymes (inhibition)	Anti-oxidant enzymes (stimulation)
D1R	NADPH oxidase, via PKA/PKC cross talk [19,45,47]	SOD, glutathione peroxidase, glutamyl cysteine transferase, and HO-1 [46,65]
D2R	NADPH oxidase [19,85,86,88]	DJ-1, PON2, and HO-2 [19,85,86] glutathione, catalase, and SOD [79]
D5R	NADPH oxidase, via PLD2 [43,49]	SOD, glutathione peroxidase, glutamyl cysteine transferase, and HO-1 [46,48]
